# Antimicrobial-drug use and methicillin-resistant Staphylococcus aureus.

**DOI:** 10.3201/eid0701.010129

**Published:** 2001

**Authors:** D L Monnet, N Frimodt-Møller


This was the first case of high-level vancomycin-resistant enterococci with a class A phenotype isolated from a person in our hospital or in Ankara, Turkey. To prevent the organism's spread, we implemented the recommendations of the Hospital Infection Control Practices Advisory Committee (5).

**Ahmet Basustaoglu,* Hakan Aydogan,* Cengiz Beyan,* Atilla Yalcin,* Serhat Unal†**
*Gülhane Military Medical Academy, Etlik Ankara, Turkey; †Hacettepe University, Ankara, Turkey

### References

1. Leclercq R, Derlot E, Duval J, Courvalin P. Plasmid-mediated resistance to vancomycin and teicoplanin in *Enterococcus faecium*. N Engl J Med 1988;319:157-61.
2. Uttley AH, George RC, Naidoo J, Woodford N, Johnson AP, Collins CH, et al. High level vancomycin-resistant enterococci causing hospital infection. Epidemiol Infect 1989;103:173-81.
3. Dutka-Malen S, Evers S, Courvalin P. Detection of glycopeptide resistance genotypes and identification of the species level of clinically relevant enterococci by PCR. J Clin Microbiol 1995;33:24-7.
4. Handwerger S, Skoble J, Discotto LF, Pucci MJ. Heterogeneity of the VanA gene clusters in clinical isolates of enterococci from the northeastern United States. Antimicrob Agents Chemother 1995;39:362-8.
5. Hospital Infection Control Practices Advisory Committee (HICPAC). Recommendations for preventing the spread of vancomycin resistance. Infect Control Hosp Epidemiol 1995;16:105.

### Antimicrobial-Drug Use and Methicillin-Resistant *Staphylococcus aureus*

**To the Editor:** We read with great interest the debate on the contribution of antimicrobial selection pressure to changes in resistance in *Salmonella enterica* serovar Typhimurium and the comparison made with methicillin-resistant *Staphylococcus aureus* (MRSA) (1).

We strongly agree with Davis et al. that infection control practices must play a central role in successful MRSA control programs. However, we disagree that the antimicrobial-drug use practices that contribute to the control of MRSA have not been scientifically defined. In a recent review, we identified more than 20 studies on consistent associations, dose-effect relationships, and concomitant variations, all supporting a causal relationship between antimicrobial-drug use and MRSA (2).

Since our review, seven other studies have reported on the contribution of antimicrobial-drug use to MRSA colonization and infection in patients, or to high MRSA rates in health-care settings (3-9). One study reports a decrease in the rate of new MRSA cases after major reduction in antimicrobial-drug use (5). Although a lower number of discharges and a shorter hospital stay recorded during the 2-year postintervention period have been proposed as other explanations (10), the sharp decrease in new MRSA cases after the new antibiotic formulary was implemented (a delay of only a few months) supports the hypothesis that reduced antimicrobial pressure contributed to the decline. Additionally, at the recent 4th Decennial International Conference on Nosocomial and Healthcare-Associated Infections, at least five reports addressed either (a) antimicrobial-drug use and increased MRSA incidence or (b) antimicrobial-drug use as an independent risk factor for MRSA acquisition or for persistent MRSA colonization after mupirocin treatment (11).

When antimicrobial classes are taken into account separately, cephalosporins and fluoroquinolones are often identified as risk factors for MRSA (2-5,8,11). The mechanisms that would explain the participation of these two classes are not fully understood. However, fluoroquinolones directly enhance the expression of high-level oxacillin-resistant *S. aureus* in vitro (11, p.202). Another recent study shows that sub-MIC levels of ciprofloxacin increase adhesion of quinolone-resistant MRSA (12), which could explain persistent MRSA colonization and failure of mupirocin treatment in patients who received a fluoroquinolone (11, p.197). MRSA outbreaks in surgical patients have been controlled by isolating patients and abandoning third-generation cephalosporins for surgical prophylaxis (3). As stated by Davis et al., dissemination of epidemic clones does not necessarily require antimicrobial selection pressure; however, the above studies suggest participation of antimicrobial drugs in MRSA colonization and outbreaks.

Finally, when citing Dutch infection control measures as an example of successful control of MRSA, Davis et al. omit the fact that, among European countries, the Netherlands has the lowest antimicrobial-drug use in primary health care (13) and one of the lowest in hospitals (14). Similarly, Nordic European countries report both very low MRSA prevalence and antimicrobial-drug use (13,15). In Denmark, the prevalence of MRSA peaked at approximately 18% among all *S. aureus* isolates (and approximately 30% among blood isolates only) at the end of the 1960s, then regularly decreased during the 10 following years. This decrease has been attributed to various interventions, including increasing awareness of hospital hygiene and an intensive campaign to teach physicians the principles of prudent antimicrobial-drug use. Indeed, the decade witnessed a decrease in the use of streptomycin and tetracycline to which these MRSA strains were resistant. However, determining the relative contribution of these interventions to the disappearance of MRSA strains from Denmark has not been possible since all were implemented at approximately the same time. Since the beginning of the 1980s, the percentage of MRSA has remained extremely low, and below 1% among blood *S. aureus* isolates. Except for a very small number of localized hospital outbreaks, Danish MRSA isolates now represent imported cases from countries with high prevalence. To preserve this low level, patients admitted from foreign hospitals are isolated and screened for MRSA carriage. Health-care workers who have been working in foreign hospitals are also screened before working in Danish hospitals. At the same time, both the overall level of antimicrobial-drug use and the fraction represented by broad-spectrum antimicrobial drugs, such as cephalosporins or fluoroquinolones, remain very low in Danish primary health care and hospitals, according to the 1999 report by the Danish Integrated Antimicrobial Resistance Monitoring and Research Programme (available from: URL: http://www.svs.dk/dk/Organisation/z/forsider/Danmap%20forsider.htm).

Additional research is certainly needed to fully understand the relationship between antimicrobial use and MRSA. However, the evidence supports implementation of programs to control or improve prescriptions when infection control alone does not control MRSA or the organization and resources for a "search-and-destroy" MRSA control strategy are not available.

**Dominique L. Monnet and Niels Frimodt-Møller**
Statens Serum Institut, Copenhagen, Denmark

### References

1. Davis MA, Hancock DA, Besser TE, Rice DH, Gay JM. Reply to Drs. Angulo and Collignon. Emerg Infect Dis 2000;6:437-8.
2. Monnet DL. Methicillin-resistant *Staphylococcus aureus* and its relationship to antimicrobial use: possible implications for control. Infect Control Hosp Epidemiol 1998;19:552-9.
3. Fukatsu K, Saito H, Matsuda T, Ikeda S, Furukawa S, Muto T. Influences of type and duration of antimicrobial prophylaxis on an outbreak of methicillin-resistant *Staphylococcus aureus* and on the incidence of wound infection. Arch Surg 1997;132:1320-5.
4. Hill DA, Herford T, Parratt D. Antibiotic usage and methicillin-resistant *Staphylococcus aureus*: an analysis of causality. J Antimicrob Chemother 1998;42:676-7.
5. Landman D, Chockalingam M, Quale JM. Reduction in the incidence of methicillin-resistant *Staphylococcus aureus* and ceftazidime-resistant *Klebsiella pneumoniae* following changes in a hospital antibiotic formulary. Clin Infect Dis 1999;28:1062-6.
6. Onorato M, Borucki MJ, Baillargeon G, Paar DP, Freeman DH, Cole CP, et al. Risk factors for colonization or infection due to methicillin-resistant *Staphylococcus aureus* in HIV-positive patients: a retrospective case-control study. Infect Control Hosp Epidemiol 1999;20:26-30.
7. Pujol M, Corbella X, Peña C, Pallares R, Dorca J, Verdaguer R, et al. Clinical and epidemiological findings in mechanically-ventilated patients with methicillin-resistant *Staphylococcus aureus* pneumonia. Eur J Clin Microbiol Infect Dis 1998;17:622-8.
8. Schentag JJ, Hyatt JM, Carr JR, Paladino JA, Birmingham MC, Zimmer GS, et al. Genesis of methicillin-resistant *Staphylococcus aureus* (MRSA), how treatment of MRSA infections has selected for vancomycin-resistant *Enterococcus faecium*, and the importance of antibiotic management and infection control. Clin Infect Dis 1998;26:1204-14.
9. Soriano A, Martínez JA, Mensa J, Marco F, Almela M, Moreno-Martínez A, et al. Pathogenic significance of methicillin resistance for patients with *Staphylococcus aureus* bacteremia. Clin Infect Dis 2000;30:368-73.
10. Rice LB. Editorial response: a silver bullet for colonization and infection with methicillin-resistant *Staphylococcus aureus* still eludes us. Clin Infect Dis 1999;28:1067-70.
11. Abstracts of the 4th Decennial International Conference on Nosocomial and Healthcare-Associated Infections, Atlanta, Georgia, March 5-9, 2000. Infect Control Hosp Epidemiol 2000;21:120-122,124,135. (http://www.slackinc.com/general/iche/stor0200/abs1.pdf for pp. 120, 121, 122, and 124; and http://www.slackinc.com/general/iche/stor0200/abs2.pdf for p. 135).

12. Bisognano C, Vaudaux P, Rohner P, Lew DP, Hooper DC. Induction of fibronectin-binding proteins and increased adhesion of quinolone-resistant *Staphylococcus aureus* by subinhibitory levels of ciprofloxacin. Antimicrob Agents Chemother 2000;44:1428-37.
13. Cars O, Mölstad S, Melander A. Large variation in antibiotic usages between European countries [abstract MoP299]. Clin Microbiol Infect 2000;6(Suppl 1):216.
14. Janknegt R, Oude Lashof A, Gould IM, van der Meer JWM. Antibiotic use in Dutch hospitals 1991-1996. J Antimicrob Chemother 2000;45:251-6.
15. European Antimicrobial Resistance Surveillance System. EARSS Newsletter. Apr 2000 (2). Available from: URL: http://www.earss.rivm.nl/PAGINA/DOC/newslapril2000_arial.pdf

### Lack of Evidence for Chloramphenicol Resistance in *Neisseria meningitidis*, Africa

**To the Editor:** High-level chloramphenicol resistance has been reported in 11 epidemiologically unrelated *Neisseria meningitidis* serogroup B strains in Vietnam and in a single strain in France, all isolated between 1987 and 1996 (1). Resistance was mediated by a chloramphenicol acetyltransferase (Cat) encoded by a *catP* gene homologous to *Clostridium perfringens* transposon Tn*4451*. While used infrequently in industrialized countries, chloramphenicol is often used to treat patients with meningococcal disease in Africa, especially during epidemics, when it frequently becomes the drug of choice because it can be administrated intramuscularly (2).

To evaluate the presence of meningococcal chloramphenicol-resistant isolates in Africa, we assessed the frequency of the *catP* gene in 33 *N. meningitidis* strains of serogroup A from the collection of the Centers for Disease Control and Prevention's Epidemic Investigations Laboratory. The isolates, selected to give the maximum geographic and chronological representation, were collected during 1963 to 1998 from Chad, Egypt, Gambia, Ghana, Niger, Nigeria, South Africa, Tanzania, and Uganda, mostly during outbreaks. Thirteen (39.3%) of the strains were isolated during the 1990s, when chloramphenicol resistance was first described in Vietnam. All isolates were characterized by multilocus enzyme electrophoresis and represented four major electrophoretic subgroups (3,4). Chloramphenicol and penicillin MICs were determined for all isolates, according to the recommendations of the National Committee for Clinical Laboratory Standards, by the broth microdilution method using Mueller-Hinton broth with 5% lysed horse blood incubated in 5% $CO_2$ (5). All isolates were susceptible to both chloramphenicol (MIC <2 µg/mL) and penicillin (MIC <0.06 µg/mL). In addition, we tested all isolates for the presence of *catP* by polymerase chain reaction (PCR) using primers A, B, C, and D (1). Primers A and B, designed from the sequence of *catP,* amplify a 300-bp fragment only in chloramphenicol-resistant isolates. Primers C and D, designed on the basis of meningococcal sequences flanking the Tn*4451*-like insertion, amplify ~1200-bp fragment in resistant isolates and ~200-bp fragment in susceptible strains. Strain LNP13947 (kindly provided by Marc Galimand) was used as a positive control.

The *catP* gene was not detected in 32 of 33 *N. meningitidis* serogroup A strains. One isolate that was negative with primers C and D tested positive with primers A and B (M2786, Nigeria, 1963), which could suggest that *catP* was present but in a different location in the meningococcal genome. However, the chloramphenicol MIC of that strain was 2 µg/mL (susceptible). Repeated attempts to sequence the A/B amplicon were not successful with either primers A and B or another set of primers internal to primers A and B, implying that only a portion of the *catP* gene was present or (even more likely, given the conserved nature of this gene) that the PCR result was a false positive.

Chloramphenicol resistance was first described in meningococcal serogroup B isolates (1), but only serogroup A strains were included in this study since A is the most prevalent serogroup in Africa. (It accounts for most epidemics in Sub-Saharan regions.) Although our small sample size limited the chances of detecting a rare event, the data suggest that chloramphenicol resistance in Africa is relatively infrequent and that chloramphenicol is still an appropriate agent to treat meningococcal disease.

The acquisition of plasmids encoding Cat, which enzymatically inactivate choramphenicol, is the most common mechanism of resistance in gram-positive and gram-negative organisms.

